# Spontaneous Subarachnoid Haemorrhage in Neurological Setting in Burkina Faso: Clinical Profile, Causes, and Mortality Risk Factors

**DOI:** 10.1155/2019/8492376

**Published:** 2019-05-09

**Authors:** Alfred Anselme Dabilgou, Alassane Drave, Julie Marie Adeline Kyelem, Lanseni Naon, Christian Napon, Jean Kabore

**Affiliations:** ^1^Neurology Department, Yalgado Ouedraogo University Hospital, Ouagadougou, Burkina Faso; ^2^Department of Medicine, Regional University Teaching Hospital, Ouahigouya, Burkina Faso; ^3^Neurology Department, Bogodogo University Teaching Hospital, Ouagadougou, Burkina Faso

## Abstract

To determine the prevalence, clinical profile, causes, and mortality risk factors of spontaneous arachnoid haemorrhage at Yalgado Ouedraogo University teaching Hospital, we conducted a 5-year retrospective study of 1803 stroke patients admitted to Neurology Department during the period from January 2012 to December 2016. During the study period, spontaneous subarachnoid haemorrhage accounted for 3.2 % of all stroke. The mean age of patients was 60 years (range 20-93 years). There was a female predominance in 55.9%. The common vascular risk factors were hypertension (79.7%) and chronic alcohol consumption (16.9%). The main symptoms were headache (76.2%), motor weakness (74.5%), and consciousness disorders (62.7%). Neurological examination revealed limb weakness in 76.2% and meningeal irritation in 47.4%. The best admission Glasgow Coma Scale score of 15 was found only in 37.3 % of patients. About 50.8% of patients were admitted to Hunt and Hess moderate grade (III) resulting in a mortality of 24.80%. The main cause of spontaneous subarachnoid haemorrhage was hypertension (77.9%). Cause could not be determined in 8.5 % of cases. The mortality rate was 37.3%. There was high mortality in patients with intraventricular haemorrhage and in patients with disturbances of consciousness. In conclusion, our study showed a poor frequency of spontaneous subarachnoid haemorrhage with high mortality. Hypertension was the most common cause of spontaneous subarachnoid haemorrhage.

## 1. Introduction

Subarachnoid haemorrhage (SAH) is a type of stroke in which bleeding occurs in the subarachnoid space alone or in conjunction with bleeding elsewhere in the central nervous system. The haemorrhage is classified as either spontaneous (nontraumatic) or traumatic. Spontaneous subarachnoid haemorrhage is due to the rupture of either an aneurysm or arteriovenous malformation or due to hypertension or an unknown cause. They represent 5% of all strokes and are half as frequent as intraparenchymal haemorrhages [1]. Ruptured intracranial aneurysm is the most common cause of spontaneous SAH (around 85% of cases) [2]. SAH is a serious condition characterized by 50% mortality and functional dependence of one-third of survivors [3]. The worldwide incidence rate of spontaneous subarachnoid haemorrhage is approximately 9.1 per 100 000 person-years but there is a lack of information about SAH incidence and outcome in populations of African descent in America [4]. In sub-Saharan African, incidence of SAH is unknown and there are few studies about SAH in African population. The objective of this study is to describe the characteristics of SAH in a hospital setting in Burkina Faso.

## 2. Materials and Methods

### 2.1. Area of Investigation

Burkina Faso is a French speaking country located in West Africa region. Its surface area is 224 000 km^2^ with a population of 16 000 000 inhabitants according to 2016 National Census. The population is served by 13 regional hospitals and 6 university teaching hospitals including Yalgado Ouedraogo Hospital. Medical care is at charge of the patients who have a limited access to hospitals, high-quality medical services, and diagnostic technologies, including CT (computerized tomography) and MRI (Magnetic Resonance Imaging). CT is available in many medical centres but MRI is available in only 2 medical centres in Ouagadougou, the capital. The University Hospital Yalgado Ouedraogo accounts Emergency Department, Resuscitation Department, Neurology Department, and Neurosurgery Department which admitted patients with subarachnoid haemorrhage. All the patients with SAH were admitted to Emergency Department and after were transferred to Neurology Department or Resuscitation Department. Only patients with aneurysmal SAH were admitted to Neurosurgery Department. Currently, there are no specific treatments for SAH aneurysmal in Burkina Faso, so the patients were transferred to Europe for treatment.

## 3. Study Design

We conducted a 5-year retrospective study from January 2012 to December 2016 at Neurology Department of Yalgado Ouedraogo University Hospital.

### 3.1. Study Population

Only patients with spontaneous subarachnoid haemorrhage were included. We included patients aged 18 years who had performed brain CT, blood count, CRP, prothrombin, blood glucose, and electrolytes test. We do not include patients without medical data.

### 3.2. Data Analysis

The subarachnoid haemorrhage grade at admission was recorded for all patients using the Glasgow Outcome Scale [2], the Hunt-Hess scale [5], and WFNS grade [6]. The analyses were carried out using the software Epi info in version 3.5.1. The averages and percentages were analyzed by Khi2, Student's tests for large averages, and Fischer's exact test for small sizes. The test is considered statistically significant for p less than 0.05. We respected the confidentiality of the information given to us.

## 4. Results

During the study period, 1803 stroke patients were admitted to Neurology Department. There were 1253 (69.5%) cases of ischemic stroke and 550 (30.5%) cases of haemorrhagic stroke. Subarachnoid haemorrhage accounts for 3.3% of all stroke (59/1803) and 10.7% of haemorrhagic stroke (59/550). There was an average of 11 SAH cases annually. The median time from onset of symptom to admission was 2.16 days (range 24h–9 days). Thirty-six (61%) patients were admitted within the first 24 hours. The mean age of patients was 60 years (range 20-93 years). The mean age for of male (57.57 years) was higher than for women (47.18 years). Almost SAH was in the group of 50 to 60 years (27.1%). Patients with age group under 40 years were 12 (20.3%) and those aged over 60 were 20 (33.8%). There were 33 female patients (55.9%) and 26 male patients (44.1%) included in the study. The majority of patients had lived in urban areas (66.1%). The common vascular risk factors were arterial hypertension in 47 (79.7%) patients, chronic alcohol consumption in 20 (33.9%) patients, and having history of stroke in 6 (10.2%) patients.  Table 1 describes distribution of patients by age group.

### 4.1. Clinical Characteristics

The main symptoms were headache in 45 (76.3%) patients motor weakness in 44 (74.6%) patients and loss of consciousness in 37 (62.7%) patients. At the diagnosis of SAH, 47 patients (79.7%) had elevated blood pressure. Most patients had severe hypertension (53.2%), grade 2 (31.9%) and grade 1 (14.9%). Hyperthermia (>38.5) was seen in 47 patients (79.7%).  Table 2 describes the main complaints of patients at admission.

### 4.2. Neurological Status on Admission

Limb weakness was present in 45 patients (76.3%) with hemiplegia in 41 patients (69.5%) and tetraplegia in 4 patients (6.8%). The meningeal irritation was present in 28 patients (47.5%). Patients without a focal neurodeficit were found in 14 patients (23.7%). Cranial nerve involvement was found in 18 (30.5%) patients. The best admission Glasgow Coma Scale score of 15 (WFNS grade of 1) was found only in 22 patients (37.3 %), while the worst GCS score of 3-6 (WFNS grade 5) was applicable to 8 patients (13.6%).

### 4.3. Hunt and Hess Grading System

About 17 patients (28.8%) of all SAHs were admitted to Hunt and Hess good grades (I; II), resulting in a mortality of 19.69%. Of SAHs with poor grades (IV; V), 12 patients (20.3%) had worst mortality of 63.15%, and of moderate grade (III) SAHs admitted, 30 (50.8%) were having a death rate of 24.80%.

### 4.4. Brain Investigations

The Noncontrast CT (NCCT) of brain was performed in all of our patients, in the mean time of 2.40 days (range 1-15 days). The initial scan showed blood on basal cisterns in 24% of patients, cortical sulcus in 41%, Sylvian fissures in 13%, and being not notified in 22%. The NCCT brain showed intraventricular haemorrhages (IVHs) in 59.3% (35/59). The intracerebral haemorrhages (ICH), lobar, thalamic, etc., were found on initial CT-scan in 42 patients (71.2%) of SAHs. The hydrocephalus was found in 10.6% (83/785) scans. The SAH of Fisher grades 2 (SAH less than 1 mm thick on CT-scan) and 3 (more than 1 mm thick) appearance was found in 18.6% (11/59) scans. The Fisher grade 4 (any thickness with intraventricular haemorrhage or parenchymal extension) appearance was found in 81.4 % (48/59) of those scanned for SAH. The NCCT brain for SAH was 100% sensitive in the study. About 14 (23.7%) patients were subjected to the CT-angiography. The CT-angiography was negative in 10 patients (71.4%). There were one case of aneurysm in internal carotid (10 mm), one case of aneurysm in cerebral anterior artery (8mm), and two cases of aneurysm in Sylvian sulcus.

### 4.5. Others Investigations

The main abnormalities were stress hyperglycemia in 61% (36/59), hyperleukocytosis in 52.5 (31/59), hypernatremia <132 mmol/l in 27.1% (16/59), and hypomagnesaemia <0.7 mmol/l in 20.3% (12/59) patients. Renal dysfunction was found in 8 (13.6%) patients with creatininemia >169 *μ*mol/l. There were thrombopenia < 100 000 in 7 (11.9%) patients and low level of prothrombin < 70% in 4 patients. The electrocardiography showed atrial fibrillation in 6 (10.2%) patients, tachycardia in 7 (11.9%), sinus bradycardia in 4 patients (6.8%), supraventricular extrasystoles, and repolarization disorders in a patient.

### 4.6. Causes

The main cause of SAH was hypertension in 46 patients (78%), followed by aneurysmal rupture in 4 (6.8%) patients and coagulation disorders in 4 patients (6.8%). There were two cases of severe thrombocytopenia (< 40 000 / *μ*l) and low level of prothrombin time activity PTA (< 25%) in 2 (3.4%) patients. Neither history nor investigations revealed any cause for 5 (8.5%) of the SAHs which were labeled as idiopathic.

### 4.7. Prognosis

The length of stay was 15 days. During hospitalization period, four patients presented with venous thromboembolism (pulmonary embolism) and two with pulmonary artery disease. During the 30 days following SAH diagnosis, 22 (37.3%) patients died. Hypertensive SAH was the most common cause of death in 81.8%, followed by idiopathic SAH (9.1%). The recovery of the motor deficit was noted in 82.9% (34/41) of patient with motor deficit. The main mortality risk factors were ventricular haemorrhage (p = 0.00032 < 0.05) and disturbances of consciousness (p = 0.0006 < 0.05).  Table 3 presents the mortality risk factors of SAH.

## 5. Discussion

In our study, SAH accounted for 3.3% of all stroke. This frequency is lower than in literature who found 5% [1]. A higher frequency was seen in the Nigerian stroke registry (11.3%) [7] and in Kashmir (31.02%) [8]. We collected 59 cases of SAH in 5-year study in a single hospital. In contrast, Wakesa in Kenya had recruited 55 patients with SAH in Kenyatta National Hospital between December 2010 and March 2011 [9]. The low number of patients in our study could be explained by the population characteristics. The mean age of patients (60 years) was in concordance with studies made in Sweden (60 years), China (59±13 years), and America (63.1± 12.6 yeas) [10–12]. In contrast, Wekesa et al. in Kenya had found a lower mean age of 47.5± 18.3 years [9]. According to literature, the peak incidence of HSA is around the 5th and 6th decades [13]. Spontaneous subarachnoid haemorrhage is most common in women [8–13]. Female gender increases 1.24 to 1.74 times the risk to develop SAH [14]. A history of hypertension was documented in the majority of cases in our context in concordance with some studies [9, 12]. Chronic alcohol consumption was present in 33.9% of cases, three times more than in USA (5.6% [12]. Atrial hypertension multiplied by 2.8 times the risk to develop SAH and excessive alcohol intake (risk multiplied by 1.5) [15, 16].

### 5.1. Clinical and Etiologic Aspects

Acute headache and severe hypertension were, respectively, seen in 76.2% and 53.2% of patients. Both are clinical characteristics of SAH [17]. The most frequent neurological symptoms was motor deficits (74.5%) followed by meningeal irritation (47.4%). Meningeal irritation was seen alone in 14 patients (23.7%). In contrast, meningeal irritation was the most common sign seen in Kashmir with 68.46% [8]. Meningeal irritation may be lacking during admission to the emergency department [18]. Loss of consciousness was seen in 62.7% of patients, consistent with literature [19]. According to etiologies, hypertensive SAH was the main cause (77.9%), in agreement with others studies [20]. Aneurysmal SAH was rare in our study (6.8%), in concordance with the study of Wakesa in Kenya with a prevalence of 29% [9]. This feature was in opposition to the literature which showed that 85% of SAH cases are due to a ruptured cerebral aneurysm [21, 22].

This situation could explain that CT-angiography or MRI-angiography was expensive for the majority of patients. Indeed, CT-angiography was negative in 71.4% of patients who done this investigation. According to literature, aneurysm is not detectable by cerebral angiography in 15% to 20% of patients with SAH [23].

### 5.2. Mortality of SAH

The mortality rate of SAH was 37.3% in our study, higher than that found in Kashmir (20.38%) [8], in Martinique (24%) [24], and in developed countries [25]. In a previous autopsy study from Kenya, 2.1% of 134 deaths were due to subarachnoid haemorrhage [26]. In Africa, between 3% and 26% of patients with SAH die prior to reaching the hospital and those who do arrive alive have a high likelihood of rapid deterioration and mortality rate of up to 33% [27]. In our study, there was relation between mortality and sex (p = 0.45). This result is in agreement with the literature [13, 15]. Loss of consciousness was significantly associated with an increased risk of death (p = 0.0006). A low neurological score (GCS < 10) is associated with a high risk of mortality [13]. Intraventricular haemorrhage and hydrocephalus were also associated with a high risk of death (p< 0.05). Intraventricular haemorrhage is a great predictor of hydrocephalus [28, 29].

## 6. Limitations

This present study has some limitations. As this study was a single hospital-based study conducted on patients belonging to lower socioeconomic status having a different clinical and risk factor profile, these results cannot be applied to the general population. As Doppler sonography was performed by radiologists and cardiologists, an observer bias in categorization of the carotid stenosis could not be ruled out.

## 7. Conclusions

This study showed a poor frequency of spontaneous subarachnoid haemorrhage with high mortality. Atrial hypertension was the most common cause of spontaneous subarachnoid haemorrhage. There was a lack of CT-angiography investigation in the majority of patients.

## Figures and Tables

**Table 1:** Distribution of patients with SAH according to age group (n=59).

Age group ( years)	Overall ( n=59)	Male (n=26)	Female (n=33)
20-30	2 (3.4%)	0	2 (6.1%)

31-40	10 (16.9%)	5 (19.2%)	5 (15.2%)

41-50	11(18.6%)	7(26.9%)	4 ( 12.1)

51-60	16 (27.1%)	8 (30.8%)	8 (24.2%)

61-70	10 (16.9%)	2 (7.7%)	8 (24.2%)

71-80	8 (13.6%)	3 (11.5%)	5 (15.1%)

>80	2 (3.4%)	1 (3.8%)	1(3%)

**Table 2:** Symptoms of SAH at admission.

Symptoms at admission	Overall (N=59)	Percentage
Headache	45	76.3

Limb Weakness	44	74.6

Loss of consciousness	37	62.7

Speech disorder	28	47.5

Vomiting	25	42.4

Agitating	11	18.6

Vertigo	10	16.9

Photophobia	2	3.4

**Table 3:** Significant prognostic indicators of mortality.

		Death		
Feature	Overall (N=59)	Yes	No	P value
*Age group (years)*				

Age ≤ 60	38	14	24	0.492

Age > 60	21	8	13	

*Gender *				

Male	25	10	15	

Female	34	12	22	0.45

*unconscienness*				

Yes	37	19	18	

No	22	3	19	0.0006

*Intraventricular haemorrhage *				

Yes	35	16	19	

No	24	6	18	0.00032

*Hydrocephalus *				

Yes	9	2	7	

No	50	20	30	0.035
